# Measuring hemophilia caregiver burden: validation of the Hemophilia Caregiver Impact measure

**DOI:** 10.1007/s11136-017-1572-y

**Published:** 2017-04-25

**Authors:** Carolyn E. Schwartz, Victoria E. Powell, Adi Eldar-Lissai

**Affiliations:** 1 DeltaQuest Foundation, Inc., 31 Mitchell Road, Concord, MA 01742 USA; 2 Departments of Medicine and Orthopaedic Surgery, Tufts University Medical School, Boston, MA USA; 3 Biogen, Cambridge, MA USA

**Keywords:** Hemophilia, Caregiver, Burden, Measure, Item response theory, Psychometrics

## Abstract

**Aims:**

The purpose of this article is to describe the psychometric development of the Hemophilia Caregiver Impact measure.

**Methods:**

Qualitative interviews (*n* = 22) and a cross-sectional web-based study (*n* = 458) were implemented with caregivers of people with hemophilia. Classical test theory and item response theory analyses were implemented to evaluate the psychometric characteristics of the measure.

**Results:**

The study sample had a mean age of 39 and a median level of college education. It was predominantly female (88%), and had an average of two children. 85% of this study sample had at least one child with hemophilia. The final 36-item Hemophilia Caregiver Impact measure is composed of seven subscales assessing relevant negative aspects of caregiver impact (Burden Summary) as well as one subscale reflecting a positive aspect of caregiver impact (Positive Emotions). These two summary scores are orthogonal and can be used together in analyses examining negative and positive aspects of caregiver impact. The items included within each subscale reflect a unidimensional construct, demonstrate good item information and trace lines, and lack of local dependence. The resulting subscales demonstrate high reliability, and good construct validity. They show moderate incremental and discriminant validity.

**Conclusions:**

The Hemophilia Caregiver Impact measure is a useful new tool for clinical research on hemophilia. In addition to having eight relevant subscales, the measure can also be summarized with two scores. This versatility can be useful in analyzing studies with very small samples, which is to be expected when dealing with a rare condition like hemophilia.

**Electronic supplementary material:**

The online version of this article (doi:10.1007/s11136-017-1572-y) contains supplementary material, which is available to authorized users.

## Introduction

Hemophilia is a sex-linked hereditary bleeding disorder caused by lack of clotting factor in the body [1]. Since people are born with this disease, family members are initiated into a caregiving role as soon as the family becomes aware of the diagnosis. Caregiving for someone with a chronic health problem can be a demanding role, requiring constant vigilance, and numerous changes to one’s lifestyle [2]. Caregiving can impact one’s employment, career path, finances, social connections, and physical health [3, 4]. It can impact family functioning, as the focus on the sick family member takes precedence over others’ needs and wishes [5]. In addition to the negative aspects of caregiving, there are positive aspects, such as providing the caregiver with a sense of purpose and self-worth [2].

Understanding and tracking caregiver burden in hemophilia can be a useful metric for understanding the disadvantages and benefits of treatments for hemophilia. If a treatment affects the management of hemophilia, it will likely not only improve the patient’s clinical profile (e.g., annual bleeding rate) but will also reduce the caregiver’s perceived burden. Such a measure could be integrated into standard hemophilia supportive-team care for targeting interventions to prevent further problems. Finally, it can be a useful outcome measure for interventions aimed directly at caregivers [6] to document the impact of supportive care over time.

While there is a large and growing literature on caregiver burden [7–9], to our knowledge there is no validated measure of hemophilia caregiver burden. Most caregiver measures are designed for use across illness groups (i.e., generic) [7, 10], or are aimed at specific illness groups that do not include hemophilia [11–13] and accordingly do not measure key domains for hemophilia caregiving (e.g., practical support needed, impact on caregiver’s time for self- and other family members’ care, burden related to the hereditary nature of the disease, impact of hemophilia symptoms on caregiver’s emotional health, the positive aspects of caregiving). The only measure of hemophilia caregiving that we found [14] had not been validated using current psychometric standards [15]. We thus sought to develop such a measure of hemophilia caregiver burden for use in clinical research. The purpose of this article is to describe the psychometric development of the Hemophilia Caregiver Impact (HCI) measure.

## Methods

### Design

#### Qualitative phase

An initial qualitative validation process for the HCI involved:

- A literature review using Ovid with search terms “caregiver,” “burden,” and “measurement.” Based on this review, we developed a conceptual model based on the caregiving literature. We also implemented an Ovid literature review with search terms “hemophila,” “quality of life,” ”treatment,” etc., to develop a background on hemophilia.
- Drafting a set of items (i.e., questions) tapping the dimensions and sub-dimensions identified as important in this conceptual model. This process involved reading existing measures and identifying items that seemed relevant to hemophilia. We then drafted new items drawing on similar themes so that the item pool reflected the relevant concepts but did not plagiarize items from other measures.
- Qualitative interviews with 22 current hemophilia caregivers. We asked participants to answer the questions in the initial item bank using an online software and then conducted one-on-one interviews with each of them.
  This was an iterative process completed over three rounds of interviews (*N* = 8, 7, and 7, respectively). The first round focused on participants’ sharing their reactions to items, so that we could learn about what items were clear and accessible, and which were confusing or offensive. For example, “burden” was offensive to several respondents. This round helped to clarify that there were subgroups of caregivers for whom different set of items might be more or less applicable (e.g., caregivers of pre-verbal children, caregivers of older children or adults). The item pool was edited in response to this feedback, and then the second round of interviews focused on further feedback to the item bank. In this round, we learned that items were needed to cover content specific to adolescents and young adults (i.e., caregiver issues when the patient was needing to become more independent), and to address spousal conflict due to caregiving demands. The order of the subdomains was modified so that the measure ended with the positive domain items. On the basis of Round Two feedback, some items were added, others re-worded, and items/domains were re-ordered further. The survey also became more tailored, beginning with questions to specify the relationship with the patient so that all future items referenced ‘your [relation] with hemophilia’ rather than ‘your person with hemophilia.’ By round three, few items were mentioned as confusing or problematic. There were, however, a large number of items, many of which were redundant with one another. To reduce the number of items, we began by examining histograms of round three’s individual item distributions. This allowed us to identify items with ceiling or floor effects and good distributions (i.e., responses spread relatively evenly across response options). We then engaged a nurse who specialized in hemophilia to identify redundant items from those with good distributions and choose the item version that seemed best to tap the underlying concept.

**Fig. 1:**
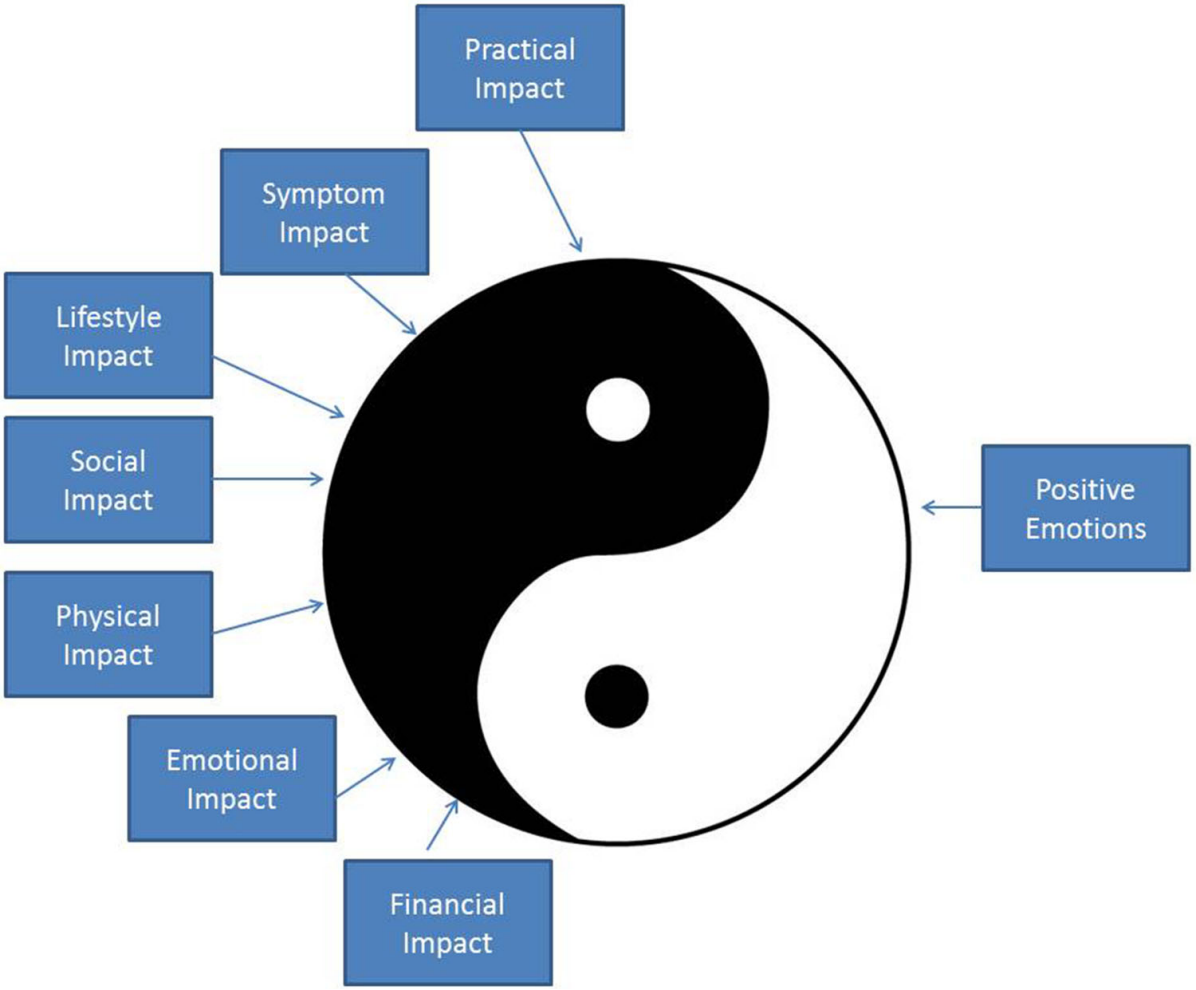
Conceptual model for the Hemophilia Caregiver Impact measure. The negative (‘yin’) and positive (‘yang’) aspects of the hemophilia caregiver experience are captured by the eight subscale domains

On the basis of these interviews, the conceptual model was honed to reflect the hemophilia caregiver experience. Figure 1 shows the final conceptual model, reflecting the ‘yin’ and ‘yang’ of the caregiver experience. The negative aspects (‘yin’) include the following impact domains: practical, symptom, lifestyle, social, physical, emotional, and financial. The positive aspect (‘yang’) comprises positive emotions, reinforcing feedback from others, and changes in their family or relationships with others that they feel reflect personal growth. These positive aspects were specifically mentioned by caregivers during the qualitative interviews as important aspects of caregiving that offset the negative aspects of burden. Without these positive aspects being assessed in the measure, they noted that we would not only have missed a critical part of the caregiving experience but also would have missed what gave value to their vigilance and efforts and enabled them to continue with equanimity. It is important to capture both the negative and positive aspects of the caregiving experience to reflect and substantiate the full experience, as well as to maximize the measure’s responsiveness to clinically important change/differences.

Additionally, these interviews assisted with refining item wording; identifying items that were not useful and therefore dropped; and identifying concepts that should be included and thus adding items. They also led to the decision to personalize the items such that it referred to “your [son/daughter/etc.] with hemophilia.” This personalization followed a question at the beginning of the survey asking to whom they were providing caregiving support. The online software auto-filled each item in the HCI measure.

The resulting item bank contained 105 items, and took an estimated 16 min to complete. These interviews provided useful information about item clarity, coverage of relevant content, and most approachable order of presentation for domains. Items and/or domains were developed as a result of this feedback. Based on these interviews, the content validity of the measure indicated that the item bank taps all the relevant domains of hemophilia caregiving.

#### Quantitative phase

A cross-sectional study was done to validate the measure and create a reliable and valid short-form version. This study collected data at baseline for the whole sample, and at one week on a random subsample for test–retest stability estimation.

### Sample

#### Eligibility criteria

Eligible study participants were caregivers of people with hemophilia A or B who were fluent in English. Only one caregiver per family was allowed to participate in the study.

#### Recruitment

Participants were recruited from several sources. We began with participants from the hemophilia panel of Rare Patient Voice, LLC., and with caregivers from the item-pretesting phase of the project. We then utilized the *snowball technique* for enhancing participant accrual. This technique involves asking study participants to refer other eligible potential participants from their network of friends and acquaintances. A natural outgrowth led to reaching out nationwide to chapters of hemophilia advocacy organizations, such as the National Hemophilia Foundation.

### Procedure

The study protocol was reviewed and approved as an exempt project by the New England Institutional Review Board (NEIRB #14-422). This web-based study was administered using the HIPAA-compliant, secure SurveyGizmo engine (www.surveygizmo.com). We followed study procedures described by Dillman’s Tailored Design Method [16] to yield a maximal response rate. Dillman’s method spells out detailed descriptions of each step of sample recruitment to yield robust response rates. It involves specific steps for personalizing study materials, providing motivating reasons for participation, paying attention to ease of use of survey interfaces, and optimal timing and content of follow-up reminders [16].

#### Incentive payments

All participants were paid $75 for participation in the baseline survey, and $35 in the one-week retest survey. We offered a $5 incentive payment to those who referred eligible study participants who then completed the survey.

### Measures

In addition to the HCI item pool (~105 items), we also collected demographic, insurance coverage, and medical/treatment information related to the hemophilia patient(s). Other person-reported measures were selected to evaluate different key aspects of validity. These measures included the *PedsQL Family Impact Module*, a generic caregiver-burden measure that contains subscales for caregiver health-related quality of life and family functioning [17]; Physical and mental health functioning were measured with the PROMIS-10 [18]. The *Ryff Psychological Well*-*Being measure subscales* for environmental mastery and social relatedness [19]; and the *Work Productivity and Activity Impairment Questionnaire* (WPAI) to assess impact of hemophilia caregiving on work [20].

### Statistical analysis

Psychometric analyses included a range of classical and modern test theory [e.g., item response theory (IRT)] analyses. We focused initially on the subset of items within a domain that explained the most variance (i.e., highest eigenvalues) in the domain (latent construct). Exploratory and confirmatory factor analyses examined the factor structure of the HCI items. Due to sample size limitations, we could not implement exploratory factor analyses on all items in one model. We thus selected items to include in domain-specific analyses initially on the basis of the abovementioned qualitative input. In some cases where an item did not exhibit good fit within a subscale, we examined its fit in another subscale that was equally credible. Sometimes this resulted in keeping the item in the other subscale, sometimes in dropping the item altogether.

On the basis of these iterative analyses, a short-form of the measure was created from a subset of the item bank, and a scoring algorithm was derived. Once unidimensional subscales were identified, graded response IRT models were computed [21]. We then examined the different dimensions of item characteristics via the IRT analyses, including item information functions, item calibrations, item thresholds, and item trace lines. Classical test theory analyses of the final short subscales included descriptive statistics to evaluate item distributions; alpha coefficients to assess internal consistency reliability; intraclass correlation coefficients (ICC) to evaluate test–retest stability; correlation analyses for construct validity assessment; polytomous logistic modeling for incremental validity assessment predicting hemophilia severity; logistic modeling for discriminant validity assessment predicting prophylaxis use; and number of people providing caregiving support (1 vs 2 or more). A second-order factor analysis investigated whether the HCI subscales could be included in one summary score for use in subsequent analyses to minimize the number of statistical comparisons.

Statistical analyses were implemented using Stata 14 [22], MPlus [23], and IRT Pro [24].

## Results

### Sample characteristics

The study sample included 458 individuals from North America, 50 of whom completed a retest survey one-week post-baseline. The sample had a mean age of 39, and a median level of education of having some college (see Table 1). The sample was predominantly female (88%), with an average of almost two children at least one of which had hemophilia. Most had private health insurance (73%), and about a quarter of the sample had government-provided insurance. Participants had been providing care to their care recipient(s) for a mean of 10.5 years, and these recipients were usually their children (90%), although ten percent of the sample provided care to other family members. The sample was predominantly Caucasian (81%); 8% were African–American, and 4% were Asian.

**Table 1:** Sample characteristics

N	458
Caregiver age	
Mean (SD)	39.24 (8.66)
Caregiver gender (%)	
Male	11.8
Female	88.0
Caregiver education (%)	
High school or less	13.8
Some college	38.7
College	31.4
Graduate degree	16.2
Insurance type (%)	
Private	73.6
Medicare, medicaid, CHAMPUS, HIS, supplemental	26.0
Does not have insurance	4.6
Missing	1.1
Number of children	
Mean (SD)	1.93 (1.20)
Number of people caring for with hemophilia (%)	
1	75.6
2	20.1
3	3.3
4	0.9
Relationship to care recipient (%)	
Son	74.2
Daughter	1.8
Children	14.2
Other family member	6.3
Multiple family members	3.3
Number of children (under age 18) with hemophilia (%)	
0	15.1
1	66.4
2	15.3
3	2.6
4	0.7
Number of years caring for patient	
Mean (SD)	10.48 (6.95)
Severity of hemophilia (%)	
Mild (%)	7.4
Moderate (%)	15.1
Severe (%)	77.1
On prophylaxis regimen (% yes)	
% yes	79.0
Race (%)	
American Indian or Alaska Native	2.8
Middle Eastern	1.1
South Asian	1.1
Other Asian	3.1
Black or African American	7.6
Native Hawaiian or Pacific Islander	0.9
Caucasian	81.0
Clotting factor products (%)	
Advate	41.1
Adynovate	1.1
Alprolix	3.7
Benefix	14.9
Eloctate	5.2
Helixate FS	3.5
Hemofil	0.2
Ixinity	0.5
Kogenate FS	9.6
Monoclate	0.4
Mononine	0.5
Novoeight	2.8
Recombinate	2.5
Rixibis	1.6
Xyntha	3.0
Other	9.1

### Psychometric development of the HCI

#### Factor analyses and IRT analyses

The first round of exploratory factor analyses (EFAs) were done using Mplus, and investigated the HCI factor structure according to presumed subscales from the qualitative phase of the work. The focus was on identifying a set of items within each domain that was unidimensional. A test-validate approach was used such that the EFAs were done on 60% of the sample so that the final subscales could be confirmed in the remaining 40% sample. This approach allows for confirming a robust factor structure.

##### Results of the round one EFAs

Eigenvalues suggested that Practical Impact, Physical Impact, and Financial Impact could be summarized by one subscale (factor) each, whereas Symptom Impact, Lifestyle Impact, Social Impact, Emotional Impact, and Positive Emotions all might be one or more subscales (factors). This decision was based either on which factors had eigenvalues greater than 1.0, or on whether there was a large difference in the eigenvalues from the first to the second factor, even if both were greater than 1.0 (e.g., 24.7 vs. 2.8). Both pieces of information might support a one-factor solution.

We then looked at the factor solutions generated and noted that the first factors within each domain-specific analysis all had strong factor loadings (i.e., greater than 0.40) and reasonable indices of model fit (Comparative Fit Index (CFI) around 0.90 or greater; Root Mean Square Error Approximation (RMSEA) around 0.11 or less). In contrast, the second factors often had double-loading items (i.e., items loaded strongly on both factors) or the second factor contained very few items and they did not seem to reflect a unified theme. It should be noted that when model fit indices did not agree, the CFI took precedence because the RMSEA is sensitive to small sample sizes [25].^1^ This is particularly apparent with the Financial subscale in this round of analyses because unemployed caregivers would have missing data on three of the six items, and on one of the items if they were not married/had no partner.

We examined whether items within one presumed subscale might actually fit another subscale reasonably well and then re-ran the EFAs with these items in both the original and the alternative subscales to see in which domain the item fit the best.

##### Results of the round two EFAs

This round of analyses used the reorganized items. This analysis was done using Mplus, and supported the unidimensionality of the eight domains: although several domains had eigenvalues greater than 1.0 for more than the first factor, the difference in eigenvalues for factor one and subsequent factors was so large that a one-factor solution would be appropriate. Further, the model fit and the factor loadings supported the idea that more than one factor (or subscale) was not necessary to capture the information for that domain.

We then implemented confirmatory factor analyses (CFAs) on the remaining 40% sample based on the second round of EFAs. Given the small sample size, the focus of the CFAs was less on model fit indices and more on item loadings to identify items that might be dropped for a short-form. These CFAs helped us to identify candidate items to be dropped on the basis of lower factor loadings relative to the other items. Before dropping these items, however, we implemented graded response IRT models for each domain using whole-sample data to investigate item characteristics. Graded response models are appropriate when item responses can be categorized as ordered categorical responses, such as those used in Likert rating scales [21]. These models contain two parameters: item discrimination and item difficulty. These analyses focused on other dimensions of item characteristics to help identify which items could be dropped for the short-form. The dimensions included item discrimination, item difficulty, local dependence, item information functions, and item trace lines (see Supplemental Text for brief description of each of these psychometric characteristics examined in the IRT analyses).

On the basis of the 40% sample CFAs followed by the whole-sample IRT analyses, we reduced the number of items within each domain’s subscale score to 3–5 items. We re-ran the CFAs and IRT analyses on the whole sample to confirm that these short-form subscales showed good model fit, strong item loadings, good item discrimination and difficulty, no local dependence, good item information, and good item trace lines. We checked that proposed subscales had high internal consistency. We examined test–retest reliability (i.e., stability) using intraclass correlation coefficients [26].

The resulting HCI measure contains eight subscales with a total of 36 items. According to the SurveyGizmo software, the measure is estimated to take 4–7 min to complete, is highly accessible (i.e., has content that is not difficult for hearing- or sight-impaired users), and is moderately fatiguing (i.e., not too long or with complicated answer options).

The final HCI item content within subscales, scoring, model fit statistics, factor loadings, and reliability coefficients are shown in Table 2. All eight subscales had high coefficients related to CFI model fit (CFI range 0.99–1.0; rule-of-thumb cut-off for CFI ≥ 0.95 [27]), and RMSEA statistics were within or close to the standard cut-off rule-of-thumb of 0.06 [27] for four of the eight subscales (see Table 2). Since CFI takes precedence over RMSEA, we are confident that the CFA model fit is acceptable for all subscales.

**Table 2:** Psychometrics of final short-form version of HCI

Subscale	Item content summary	CFI	RMSEA (90% confidence interval)	Factor loading	Score range	Alpha reliability	Test–retest stability
Practical Impact	Ordering supplies, preparing medication	1.00	0.00	0.70	37.2–80.5	0.78	0.67
	Medical appointments	(0.000, 0.000)		0.85			
	Travel to hospital			0.81			
Symptom Impact	Witness pain	0.99	0.08	0.76	28.0–68.9	0.81	0.68
	Worry about pain during infusions	(0.041, 0.115)		0.58			
	Worry about bleeding pain			0.88			
	Suffer when see pain			0.83			
	Distressed with breakthrough bleeding			0.62			
Lifestyle Impact	Not enough time for self	1.00	0.04	0.92	36.9–73.9	0.90	0.74
	Stressed	(0.000, 0.108)		0.93			
	Give up exercise			0.84			
	Family gives up things			0.79			
Social Impact	Worry about family impact	0.99	0.21	0.77	38.1–79.8	0.89	0.79
	Limited time for other family members	(0.159, 0.268)		0.86			
	Stressed as a family			0.89			
	Strain with spouse/partner			0.83			
Physical Impact	Fatigue	0.99	0.06	0.91	39.0–77.5	0.93	0.9
	Sleepless nights	(0.025, 0.103)		0.90			
	Tired emotionally and physically			0.93			
	Appetite changes			0.89			
	Health suffered			0.86			
Emotional Impact	Ups and downs	1.00	0.02	0.88	37.7–76.1	0.91	0.89
	Lost control	(0.000, 0.071)		0.90			
	Always on edge			0.89			
	Stress overwhelming			0.78			
	Impending doom			0.87			
Financial Impact	Financial burdens on family	1.00	0.08	0.72	37.9–73.1	0.88	0.78
	Interfere with job or daily activities	(0.044, 0.118)		0.86			
	Lost time from work			0.88			
	Take turns going to work						
	Cut down work hours			0.84			
Positive Emotions	Feel better about self	1.00	0.10	0.73	19.6–62.2	0.88	0.8
	More compassionate	(0.067, 0.139)		0.66			
	Stronger person			0.94			
	Inner strength			0.97			
	Sense of perspective			0.83			

Response options: 5 = all of the time, 4 = most of the time, 3 = some of the time, 2 = a little of the time, 1 = none of the time, −99 = not applicable/prefer not to answer. *Scoring* for all subscales except Financial Impact is the mean of the non-missing items if no more than one item is missing. Scoring for the Financial Impact subscale is the mean of all non-missing items, with no constraints on the number of allowable missing items. This is to ensure that a Financial Impact subscale estimate is possible even when the caregiver is not working and/or married/with a partner. Interpretation: Higher scores on all but the Positive Emotions subscales indicate worse burden; on the Positive Emotions subscale, higher scores indicate more positive aspects of caregiving

#### Reliability

All subscales had high alpha reliability coefficients (Table 2) and high test–retest stability. Although Practical Impact and Symptom Impact showed lower albeit acceptable stability over one week (ICC = 0.67 and 0.68, respectively), the Burden Summary score showed high stability (ICC = 0.90). Results of analyses of these two aspects of reliability support the robustness of the HCI.

#### Scoring the HCI

Subscale scores were computed as the average of subscale items unless more than one item is missing. We made an exception for the Financial Impact subscale, allowing a mean item score to be used with whatever items were available: since three of the five items relate to work-related impacts of hemophilia care, including one that relates to having a spouse or partner, many of our caregivers had missing item data because the item was not applicable to them. Making this exception for the Financial Impact subscale enabled us not to lose the 110 participants who had missing data on any or all of these three items. Finally, the subscale scores were standardized to have a mean of 50 and a standard deviation of 10. Standardized scoring is consistent with other widely used measures (e.g., SF-36™), and is preferable because it facilitates interpretation: the mean and standard deviation are known so it is easy to understand sample characteristics.

#### Creating a Burden Summary score

A second-order factor analysis was done to evaluate whether the eight subscales could be effectively summarized by one overall score. Two factors emerged (eigenvalues 4.9 and 1.0, respectively), with all subscales but Positive Emotions loading highly on the first, and Positive Emotions loading highly on the second (see Supplemental Table 1). The Burden Summary score was created by summing the Practical Impact, Symptom Impact, Social Impact, Physical Impact, Emotional Impact, Financial Impact, and Lifestyle Impact scores and standardizing them to have a mean of 50 and a standard deviation of 10. The resulting Burden Summary scores ranged from 33.5 to 76.8, with higher scores indicating worse burden. The Positive Emotions score ranged from 19.6 to 62.2, with higher scores indicating more positive aspects of caregiving (see Table 2 for score ranges on all subscales). We worked with a Burden Summary and Positive Emotions subscale scores in subsequent analyses.

#### Construct validity of the HCI

Pearson correlation coefficients were computed to evaluate the associations among HCI subscales and between each HCI subscale and other patient-reported outcomes included in the study. Supplemental Table 2 shows the HCI inter-correlations, with the correlation coefficients color-coded by Cohen’s effect size [28]. The HCI inter-correlations suggest that the subscales generally measure related but non-overlapping constructs, with moderate inter-correlations among the negative burden-related aspects of caregiver impact (see Supplemental Table 1). The only exception is the correlation between Physical Impact and Emotional Impact, with a correlation coefficient of 0.81. We believe that this large correlation reflects the fact that the physical symptoms tapped by the Physical Impact items are symptoms that also may reflect mental health issues such as depression or anxiety (e.g., fatigue, sleep problems, appetite changes). While this high correlation might suggest that one could choose to keep one or the other subscale, keeping both subscales allows these significant symptoms to have more weight in the summary score. Positive Emotions had low to near-zero correlations with the other HCI subscales, suggesting that this subscale measures a distinct construct.

Table 3 shows the color-coded correlations among HCI scores and other patient-reported outcomes. These correlations support the construct validity of the measure because they illustrate that the subscales had small correlations with unrelated constructs; moderate correlations with subscales that assess related but not overlapping constructs, and large correlations between indicators of overlapping constructs. For example, the HCI subscales generally had expected small correlations with the PROMIS Physical and Mental Health subscales and with the Ryff well-being subscales. They had expected moderate correlations with the PedsQL subscales and with the WPAI percent overall work-impairment-due-to-health scale. HCI Symptom Impact subscale had expected small correlations with the PedsQL Physical, Emotional, Social, and Cognitive Functioning subscales; and with the Communication, Daily Activities, and Family Relationships subscales. The HCI Positive Emotions subscale had expected small or close-to-zero correlations with the PedsQL, PROMIS, Ryff, and WPAI subscales, suggesting that this HCI subscale measures a construct that is distinct from all of the PedsQL, PROMIS, Ryff, and WPAI scores. In summary, the HCI subscales measure constructs that are relevant to but distinct from family functioning, health-related quality of life, well-being, and work impairment.

**Table 3:** Construct validity correlations

Bolded values indicate correlations hypothesized to be largest

*Mean of non-missing financial items

In contrast, the HCI Burden Summary had the largest correlations with the PedsQL subscales measuring similar aspects of burden (r range = −0.66 to −0.82 for all subscales but Communication). This pattern of correlations supports the construct validity of the HCI. While this pattern of correlations suggests that the HCI and PedsQL summary scores measure overlapping constructs, the content of the HCI subscales will likely provide useful information related to hemophilia-specific caregiver burden. This hypothesis was then tested in subsequent analyses.

#### Incremental validity

Polytomous logistic regression modeling was used to examine how much the HCI Burden Summary and Positive Emotions scores complemented the PedsQL Parent HRQL (Physical, Emotional, Social, and Cognitive Functioning); Family Functioning (Daily Activities and Family Relationships); and Total scores (average of all 8 subscales) in predicting hemophilia severity. This analysis evaluates whether the HCI scores provide additional information to the PedsQL scale scores.

**Fig. 2:**
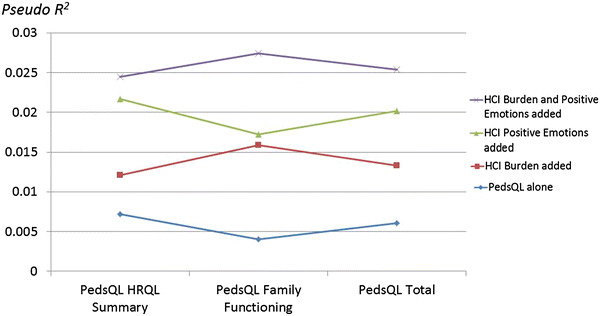
Evidence for incremental validity. The pseudo-*R* ^2^ is shown for hierarchical models explaining hemophilia severity. The PedsQL alone explained the least variance. Explained variance increased when the Burden Summary score was added, and when the Positive Emotions score was added. It was highest when both the Burden Summary and Positive Emotions scores were added to model along with the PedQL score

Figure 2 shows the comparison in estimated explained variance (pseudo *R* ^2^) in predicting hemophilia severity when the PedsQL summary scores were entered alone; when the Burden Summary was added; when the Positive Emotions subscale was added; and when both Burden Summary and Positive Emotions subscale were added. This figure illustrates that the HCI explains more variance than the PedsQL subscales alone, yielding a total of about 2% more explained variance than the PedsQL subscales alone, which explain less than 1% of the variance in severity. This added value is despite the high correlation of the HCI Burden Summary and PedsQL Total scores. Of interest, the final models suggest that Positive Emotions had independent predictive value in predicting severe but not moderate hemophilia, with a relative risk ratio of about 1.04 in the final models (*p* < 0.05) after adjusting for PedsQL scores and Burden Summary (see Supplemental Table 3).

#### Discriminant validity

Results of univariable logistic regression models predicting prophylaxis (i.e., one or more of care recipients on prophylaxis versus not) revealed that several HCI subscales were able to distinguish theses known groups (see Supplemental Table 4a). Lifestyle Impact and Positive Emotions were significantly associated with this grouping variable (*p* < 0.05 in both cases), with caregivers’ high scores on each subscale predicting caring for a patient on prophylaxis (OR = 1.03 and 1.02, respectively). Financial Impact was a trend predictor (OR = 1.02, *p* < 0.10), with caregivers’ high Financial Impact scores associated with a patient being on prophylaxis. These results make sense because being on prophylaxis requires regular, possibly daily involvement of the caregiver, which is a higher lifestyle impact. Because the patient’s health is in better control, the positive emotions aspects of caregiving would be more accessible, rather than being overshadowed by the crisis-oriented approach of on-demand therapy. Finally, prophylaxis requires regular access to factor products, which would be more costly in the short-term (i.e., more costly to the family), although more cost-effective in the long term (i.e., avoiding emergency care).

Results of a univariable logistic model predicting number of persons with hemophilia being cared for (1 vs 2 or more hemophilia care recipients) revealed a significant association of Practical Impact, with higher levels of Practical Impact associated with caring for more people with hemophilia (OR = 1.02, *p* < 0.05). There was a trend association such that higher Financial Impact and lower levels of Positive Emotions predicted caring for more people with hemophilia (OR = 1.02 and 0.98, respectively; *p* < 0.10 in both cases; see Supplemental Table 4b). These results make sense because caring for more people with hemophilia would entail more preparation of medical supplies, more medical visits, and other aspects of care measured by the Practical Impact subscale. It would also be more costly to the family, and more personally draining, making it harder to access the possible positive emotions associated with caregiving. These findings provide preliminary support to the discriminant validity of the HCI.

## Discussion

We developed an accessible 36-item Hemophilia Caregiver Impact measure composed of seven subscales assessing relevant negative aspects of caregiver impact and one subscale reflecting a positive aspect of caregiver impact. The subscales are unidimensional, demonstrate good item information and trace lines, and lack of local dependence. They demonstrate high reliability, good construct validity, and moderate incremental and discriminant validity. The positive aspects of caregiving—enhanced purpose in life, growing stronger as a person, etc.—come into play in particular for caregivers of patients with severe hemophilia.

This study has notable strengths. To our knowledge, it is the largest study of hemophilia caregivers done to date that was recruited from various sources, including the rare-disease panel and various hemophilia societies across the country. This large sample size allowed a more sophisticated set of analyses, specifically the test-validate series of IRT analyses implemented. It is also likely that the large sample size and varied recruitment strategy provide a good representative sample of hemophilia caregivers in North America. The limitations of the present work should be acknowledged. First, this is a cross-sectional study and thus not able to address the responsiveness of the HCI. Future work might collect longitudinal data from hemophilia caregivers to assess the HCI’s sensitivity to clinically important change. Second, the incremental and discriminant validity analyses provide only preliminary support for these two aspects of validity. In both sets of analyses, the amount of explained variance in outcomes examined is very small, despite the fact that the HCI subscales are statistically significant predictors of these outcomes. It is notable that the dependent variables for these analyses were all highly skewed. Future work might examine less skewed and more direct measures of caregiver burden to test incremental and discriminant validity. A better test of incremental validity might focus on a direct physiological measure of stress, such as cortisol [29]. A better discriminant validity test might compare known groups that were distinct in term of the *caregiver’s* condition rather than their caregiving context. One might compare caregivers who have regular stress-reducing activities in their life (e.g., exercise, support group) versus those without such access.

The HCI was developed using an online survey engine that supported a tailored version of the measure that allowed us to specify the relationship of the caregiver with the caregiver recipient (i.e., ‘your son with hemophilia’ rather than ‘your person with hemophilia’). This approach made the items less awkward and feel more personal. If, however, one wished to use a paper-and-pencil version of the measure, one could either revert to ‘your person with hemophilia’ or have pre-printed forms for sons, for daughters, or other common relationships.

In summary, the HCI provides a useful new tool for clinical research on hemophilia. Better treatment options would be expected to have an impact not only on the patient’s quality of life and well-being, but also would be expected to have an impact on the caregiver. In addition to having eight relevant subscales, the HCI can also be summarized with two scores: a Burden Summary score and a Positive Emotions score. This versatility can be useful in analyzing studies with very small samples, which is to be expected when dealing with a rare condition like hemophilia.

## Electronic supplementary material

Below is the link to the electronic supplementary material.

Supplementary material 1 (PDF 215 kb)

Supplementary material 2 (DOCX 18 kb)

## Footnotes

1 There is a trade-off between sample size and model size (i.e., # of parameters being estimated). That is, the denominator of the RMSEA is such that the value of the RMSEA decreases as there are more degrees of freedom (i.e., greater parsimony or fewer parameters to estimate) OR the sample size is large, keeping everything else constant. The sample size for these analyses is fairly sizable since domain-specific analyses were done separately.
