# Supplementary material for: Measuring hemophilia caregiver burden: validation of the Hemophilia Caregiver Impact measure
Source: Qual Life Res. 2017 Apr 25;26(9):2551–62. doi: 10.1007/s11136-017-1572-y (PMC5548839; doi:10.1007/s11136-017-1572-y)
Supplement: Supplementary file 1 — Supplementary material 1 (PDF 215 kb) [file 11136_2017_1572_MOESM1_ESM.pdf]

Supplemental Table 1: Second-order factor analysis for HCI subscales.

Subscale	Factor1	Factor2
Practical Impact	0.778	
Symptom Impact	0.6594	
Social Impact	0.8835	
Physical Impact	0.8926	
Emotional Impact	0.8794	
Financial Impact	0.8044	
Lifestyle Impact	0.8938	
Positive Emotions		0.9788

Supplemental Table 2 HCI Intercorrelations

	Practical Impact	Symptom Impact	Social Impact	Physical Impact	Emotional Impact	Financial Impact	Lifestyle Impact	Positive Emotions
Symptom Impact	0.5135	1						
Social Impact	0.6075	0.4443	1					
Physical Impact	0.6035	0.505	0.7869	1				
Emotional Impact	0.6113	0.5046	0.7908	0.811	1			
Financial Impact	0.5863	0.4644	0.6876	0.6916	0.6395	1		
Lifestyle Impact	0.6461	0.5184	0.7783	0.7849	0.742	0.6935	1	
Positive Emotions	-0.1106	-0.0648	-0.1388	-0.0533	-0.1309	-0.0117	-0.0942	1

small correlation: unrelated constructs (0.10 <Pearson's R < 0.40)

moderate correlation: related but not overlapping constructs (0.50 <Pearson's R < 0.80)

large correlation: overlapping constructs (0.80 < Pearson's R)

Supplemental Table 3: Incremental Validity Analyses
Polytomous logistic regression predicting severity*

Model	Severity	Predictor	RRR*	Std. Error	P value	Pseudo R2	N
1	Moderate					0.01	456
		PedsQL Parent HQRL Summary Score	0.98	0.01	0.09		
	Severe						
		PedsQL Parent HQRL Summary Score	0.98	0.01	0.09		
2	Moderate					0.00	455
		PedsQL Family Functioning Score	0.99	0.01	0.17		
	Severe						
		PedsQL Family Functioning Score	0.99	0.01	0.46		
3	Moderate					0.00	456
		PedsQL Total Score	0.98	0.01	0.10		
	Severe						
		PedsQL Total Score	0.99	0.01	0.17		

Model	Severity	Predictor	RRR	Std. Error	P value	Pseudo R2	N
1	Moderate					0.01	428
		PedsQL Parent HQRL Summary Score	0.98	0.02	0.14		
		Burden Summary	0.99	0.04	0.89		
	Severe						
		PedsQL Parent HQRL Summary Score	0.98	0.01	0.30		
		Burden Summary	1.01	0.03	0.85		
2	Moderate					0.01	427
		PedsQL Family Functioning Score	0.98	0.02	0.33		
		Burden Summary	1.01	0.04	0.76		
	Severe						
		PedsQL Family Functioning Score	1.00	0.01	0.73		
		Burden Summary	1.04	0.03	0.20		
3	Moderate					0.01	428
		PedsQL Total Score	0.97	0.02	0.18		
		Burden Summary	0.99	0.04	0.85		
	Severe						
		PedsQL Total Score	0.99	0.02	0.61		
		Burden Summary	1.02	0.04	0.62		

p< 0.05 p< 0.10

Model	Severity	Predictor	RRR	Std. Error	P value	Pseudo R2	N
1	Moderate					0.02	452
		PedsQL Parent HQRL Summary Score	0.98	0.01	0.09		
		Positive Emotions	1.01	0.02	0.71		
	Severe						
		PedsQL Parent HQRL Summary Score	0.98	0.01	0.07		

Supplemental Table 3: Incremental Validity Analyses
Polytomous logistic regression predicting severity*

		Positive Emotions	1.04	0.02	0.05		
2	Moderate					0.02	451
		PedsQL Family Functioning Score	0.99	0.01	0.16		
		Positive Emotions	1.01	0.02	0.70		
	Severe						
		PedsQL Family Functioning Score	0.99	0.01	0.34		
		Positive Emotions	1.04	0.02	0.06		
3	Moderate					0.02	452
		PedsQL Total Score	0.98	0.01	0.10		
		Positive Emotions	1.01	0.02	0.71		
	Severe						
		PedsQL Total Score	0.98	0.01	0.12		
		Positive Emotions	1.04	0.02	0.05		

Model	Severity	Predictor	RRR	Std. Error	P value	Pseudo R2	N
1	Moderate					0.02	426
		PedsQL Parent HQRL Summary Score	0.98	0.02	0.14		
		Positive Emotions	1.01	0.02	0.51		
		Burden Summary Score (financial mean)	1.00	0.04	0.92		
	Severe						
		PedsQL Parent HQRL Summary Score	0.98	0.01	0.27		
		Positive Emotions	1.04	0.02	0.04		
		Burden Summary Score (financial mean)	1.01	0.03	0.78		
2	Moderate					0.02	425
		PedsQL Family Functioning Score	0.98	0.02	0.33		
		Positive Emotions	1.01	0.02	0.51		
		Burden Summary Score (financial mean)	1.01	0.04	0.73		
	Severe						
		PedsQL Family Functioning Score	1.00	0.01	0.78		
		Positive Emotions	1.04	0.02	0.05		
		Burden Summary Score (financial mean)	1.05	0.04	0.18		
3	Moderate					0.02	426
		PedsQL Total Score	0.97	0.02	0.18		
		Positive Emotions	1.01	0.02	0.51		
		Burden Summary Score (financial mean)	0.99	0.04	0.88		
	Severe						
		PedsQL Total Score	0.99	0.02	0.57		
		Positive Emotions	1.04	0.02	0.05		
		Burden Summary Score (financial mean)	1.02	0.04	0.57		

p< 0.05 | p< 0.10

** Mild severity is the reference group; RRR= Relative Risk Ratio.*

Supplemental Table 4a: Discriminant Validity Analyses
Univariable logistic regressions using subscale scores
to predict use of prophyaxis by the index patient*

Dependent Variable: Being on prophylaxis regimen (0=not on prophylaxis, 1= on prophylaxis)

Predictor	Odds Ratio	Std. error	P value	N	Pseudo R squared
Practical Impact	1.00	0.01	0.75	455	0.00
Symptom Impact	0.99	0.01	0.42	443	0.00
Social Impact	1.02	0.01	0.16	454	0.00
Physical Impact	1.01	0.01	0.25	455	0.00
Emotional Impact	1.01	0.01	0.27	457	0.00
Lifestyle Impact	1.03	0.01	0.04	456	0.01
Financial Impact*	1.02	0.01	0.07	455	0.01
Positive Emotions	1.02	0.01	0.04	454	0.01
Burden Summary	1.01	0.01	0.24	430	0.00

Supplemental Table 4b: Univariable logistic regressions using subscale scores to predict number of people they are caring for.

Dependent Variable: Number of people with hemophilia for whom providing caregiving support (0=1 person with hemophilia, 1= more than one person with hemophilia)

Predictor	Odds Ratio	Std. error	P value	N	Pseudo R squared
Practical Impact	1.02	0.01	0.03	455	0.01
Symptom Impact	1.01	0.01	0.23	443	0.00
Social Impact	1.01	0.01	0.32	454	0.00
Physical Impact	1.01	0.01	0.40	455	0.00
Emotional Impact	1.01	0.01	0.36	457	0.00
Lifestyle Impact	1.01	0.01	0.31	456	0.00
Financial Impact*	1.02	0.01	0.10	455	0.01
Positive Emotions	0.98	0.01	0.07	454	0.01
Burden Summary	1.02	0.01	0.17	430	0.00

p< 0.05 p<0.10

*These analyses use the WHOLE SAMPLE. *Financial Impact score was the mean of non-missing items.*
