# Supplementary material for: Measuring hemophilia caregiver burden: validation of the Hemophilia Caregiver Impact measure
Source: Qual Life Res. 2017 Apr 25;26(9):2551–62. doi: 10.1007/s11136-017-1572-y (PMC5548839; doi:10.1007/s11136-017-1572-y)
Supplement: Supplementary file 2 — Supplementary material 2 (DOCX 18 kb) [file 11136_2017_1572_MOESM2_ESM.docx]

**Supplemental Text**

**Following is a brief description of each psychometric characteristic examined in the IRT analyses.**

1. **Item discrimination** provides information about how well an item separates respondents with trait levels below (to the left of) the item location from those with trait levels above (to the right of) the item location ([29](file:///C:\Users\0012579\Desktop\Schwartz%20HCI%20Validation%20Qual%20Life%20Res%20ms_rev%20final.docx#_ENREF_29)). It is shown by the steepness of the item difficulty slope (the ‘a’ parameter in the Graded Model Item Parameter Estimates table of output). Values greater than 1.0 are acceptable.
2. **Item difficulty** describes where the item functions along the trait continuum ([29](file:///C:\Users\0012579\Desktop\Schwartz%20HCI%20Validation%20Qual%20Life%20Res%20ms_rev%20final.docx#_ENREF_29)) (the ‘b_1-4_’ parameters in the Graded Model Item Parameter Estimates table of output). Items with low difficulty function among individuals with low levels of the trait being measured ([29](file:///C:\Users\0012579\Desktop\Schwartz%20HCI%20Validation%20Qual%20Life%20Res%20ms_rev%20final.docx#_ENREF_29)). High difficulty items function among individuals with high levels of the trait being measured ([29](file:///C:\Users\0012579\Desktop\Schwartz%20HCI%20Validation%20Qual%20Life%20Res%20ms_rev%20final.docx#_ENREF_29)). The difficulty index is thus a location index along the x-axis (latent trait).
3. **Local dependence.** An assumption of IRT models is that items are locally independent, meaning that no further relationship exists among items when model parameters are controlled (i.e., a person’s trait levels and item difficulties) ([30](file:///C:\Users\0012579\Desktop\Schwartz%20HCI%20Validation%20Qual%20Life%20Res%20ms_rev%20final.docx#_ENREF_30)). When items are locally dependent, they are so highly correlated that they do not bring distinct information to the score. Items with issues of local dependence are shown in red font in the Marginal Fit table of output. If two items exhibited local dependence, we would examine other item characteristics listed below to decide which of the two should be dropped.
4. **Item information functions** are shown in the Item Information Function Values table, and are shown graphically in the next page of the output. These curves show how well each level of the latent trait is being estimated by the item ([29](file:///C:\Users\0012579\Desktop\Schwartz%20HCI%20Validation%20Qual%20Life%20Res%20ms_rev%20final.docx#_ENREF_29)). A good item information curve has a bell shape indicating substantial information (y-axis) across much of the latent trait values (x-axis). Flat item information curves are indicative of items that do not function well and could be dropped.
5. **Item trace lines** show how each response option functions within an item. Ideally, item trace line graphs would have five bell-shaped curves with peaks separated across the latent trait (x-axis). This configuration indicates that each response option has a higher probability of response at distinct levels of the latent trait. Flat item trace lines indicate that the item responses do not differentiate levels of the latent trait.
